# Living with a rare disease - experiences and needs in pediatric patients and their parents

**DOI:** 10.1186/s13023-023-02837-9

**Published:** 2023-08-11

**Authors:** Stefanie Witt, Katharina Schuett, Silke Wiegand-Grefe, Johannes Boettcher, Julia Quitmann

**Affiliations:** 1 Department of Medical Psychology, Center for Psychosocial Medicine, University Medical Center Hamburg-Eppendorf, Martinistraße 52 W 26, Hamburg, 20246 Germany; 2 Department of Child and Adolescent Psychiatry, Psychosomatics and Psychotherapy, University Medical Center Hamburg-Eppendorf, Hamburg, Germany; 3 Faculty of Business and Social Sciences, University of Applied Sciences Hamburg, Hamburg, Germany

**Keywords:** Pathway to care, Psychosocial care, Pediatric patients, Parents, Rare chronic health conditions, Rare diseases, Qualitative study

## Abstract

**Background:**

A rare disease (RD) diagnosis and therapy can affect the family’s quality of life and mental health. A lack of information and missing care options lead to helplessness and psychological stress within families. This work aims to identify patients’ and parents’ experiences in daily life and with the health care system as well as their needs and current pathways to psychosocial care to develop implementation strategies adapted to the families’ needs.

**Methods:**

The present analysis is part of the national multicenter study “Children Affected by Rare Disease and Their Families-Network (CARE-FAM-NET).“ We conducted semi-structured telephone interviews with children, adolescents, and young adults with RD (aged 12 to 21 years) and parents of children with RD (aged 0 to 17 years). We analyzed the transcribed and anonymized interviews using the method of focused interview analyses to identify previous experiences with medical and psychosocial care and possible needs for improvement and support.

**Results:**

Seventy-four parents of children with RD and 15 children, adolescents, and young adults with RD participated. Five main themes emerged. *Daily life with an RD*: RD affects the everyday and social life of the respondents, negatively impacting mental well-being. *Experiences with the health care system*: The long diagnostic path is stressful for families. Professionals’ lack of information/education leads to inadequate care for those affected. *Psychosocial support*: Families do not know about psychosocial care services. In some cases, the families take advantage of psychosocial support services (such as support groups or advocacy groups), which are predominantly very helpful. *Difficulties and barriers*: Time, socio-legal and organizational problems burden families and lead to advantages in using psychosocial services. *Improvements for patient-oriented support*: Those affected wished for timely, preventive support (especially in administrative and socio-legal matters) and education regarding psychosocial care services.

**Conclusion:**

RD represent a great challenge for all family members – patients, parents, and siblings. The patients’ and parents’ previous experiences in daily life, medical and psychosocial care show a need for target-group specific support, including training of health care professionals and low-threshold access care services and practical help for all family members.

## Background

In Germany, around 2.4 to 5 million people are affected by a rare disease (RD) [1]. The specific diseases are rare, but they comprise a considerable proportion of all known diseases at around 20-26.6% [1]. In most cases, RD are congenital. As a result, around 80% of those affected by RD are pediatric patients [2]. Due to better clarification of the genetic causes of diseases, around 150 to 250 more RD are identified yearly [3, 4]. At the same time, medical health care is constantly improving, resulting in higher survival rates for pediatric patients with RD [5]. RD form a heterogeneous group of diseases and are mostly chronic. Therefore, diagnosing is often complex and takes a long time from the first symptoms to the initial diagnosis [6]. Because most RD cannot be cured or can only be treated inadequately, it strains the (pediatric) patients and their families [1].

This problematic diagnosis and treatment process can significantly burden the patients and their families [1]. The effect of the RD on the patient’s mental health and health-related quality of life (HRQOL) depends on various factors. The age and gender of the child, the severity level of the disease, the social environment, and other aspects can influence the HRQOL [7–10]. Studies report that children with RD show lower mental health than their healthy peers [11, 12]. Children and adolescents with RD show symptoms of depression, anxiety, and behavioral problems more often than healthy peers. In addition, psychological comorbidities can develop through traumatic experiences related to the disease and its treatment [13]. Above all, aspects of the progression of the disease harbor many uncertainties. As a result, children, adolescents, and young adults with RD are burdened with worries and fears about their future [14]. In particular, coping with everyday life is a massive task for the affected children and adolescents. Due to physical and sometimes mental limitations, they are dependent on the help and care of others (mostly their parents). In addition, medication or dependence on medical supply devices accompanies the daily life of children and adolescents with RD [15]. Therefore, compared to healthy peers, it is more difficult for children and adolescents with RD to develop independence and participate in social life [16].

Because RD manifest primarily in childhood, the parents take on care tasks for the affected child. Parents may be mentally stressed because of the lengthy diagnostic process and the search for treatment options. The shock of the initial diagnosis, sorrows about the progression of the disease, and the increased amount of care required to further efforts and stress for the parents of children with RD [17, 18]. Accordingly, parents of children with RD report a lower HRQOL due to posttraumatic stress disorder, loneliness, and more stress than parents of children without a disease [5, 19–22]. In addition to the day-to-day tasks and burdens of being a parent, the parents also have to cope with the demands of the child’s disease [23, 24]. They must reorganize their lives and integrate the new needs and stresses into everyday life [25]. Furthermore, the care and supervision of children with RD require much time, such as additional medical appointments and long journeys to the hospital. As a result of this additional effort, the parents - primarily the mothers - reduce their working hours or participation in social life [5]. Therefore, this exposure can limit parental HRQOL and negatively affect the parent-child relationship and child development [5, 19, 26].

Although the families feel psychologically and socially stressed by the tasks and difficulties associated with RD, parents of children with RD often do not use psychosocial offers for themselves or their children.

Psychosocial support includes interventions to preserve and improve mental health and social aspects that influence the psyche and promote psychosocial well-being. These offers aim to reduce mental illness and intend to gain coping strategies. Therefore a wide range of offers is included, such as psychotherapy, liaison services, different kind of counseling, as well as the support of and contact with patient organizations.

However, parents of children with RD either do not know about psychosocial care or do not have the time to do so [27]. In addition, most offers’ “come structure” hinder low-threshold access [28]. The parents must actively search for care offers and raise expenses such as travel, time capacities, or costs [29–31]. On the other hand, routinely offering psychosocial support directly when making the diagnosis would help the parents come to terms with the stroke of fate they experienced and the associated stress right from the start [27, 32]. Although there is contact with physicians due to the child’s regular medical visits, few psychosocial care offers are available in the clinics. Thus, although the families’ needs for care can be determined in the clinics, there is a lack of resources for adequate treatment [33].

Therefore, we aimed to identify the needs, obstacles, and barriers in families with children diagnosed with an RD from patients’ and parents’ perspectives. Based on these results, we will formulate recommendations for action in practice and research to improve the psychosocial care of families with children diagnosed with a rare chronic health condition as needed.

## Methods

### Study design

The present analysis is part of the national multicenter study “Children Affected by Rare Disease and Their Families-Network (CARE-FAM-NET)” [34]. This randomized-controlled study aims to improve and implement psychosocial care for children, adolescents, and young adults with RD and their families [34]. In addition to the main study, we aim to identify the patients’ and parents’ current experiences in daily life and with the health care system and families’ needs and pathways to psychosocial care. To comprehensively understand the patients’ and parents’ perspectives, we conducted qualitative telephone interviews with pediatric patients aged 8 to 21 and parents of pediatric patients aged 0 to 17. These telephone interviews are based on a semi-structured interview guideline. We developed the interview guideline using the results of a literature review conducted at the project’s beginning and professionals’ experiences in rare pediatric diseases. We aimed to ensure that the guideline includes all relevant topics. The semi-structured guideline enables the interviewees to name additional relevant aspects beyond the questions. In addition, the interviewer asks whether any crucial aspects have not been mentioned so far and invites the interviewee to add this information and experiences at the end of the interview.

**Fig. 1:**
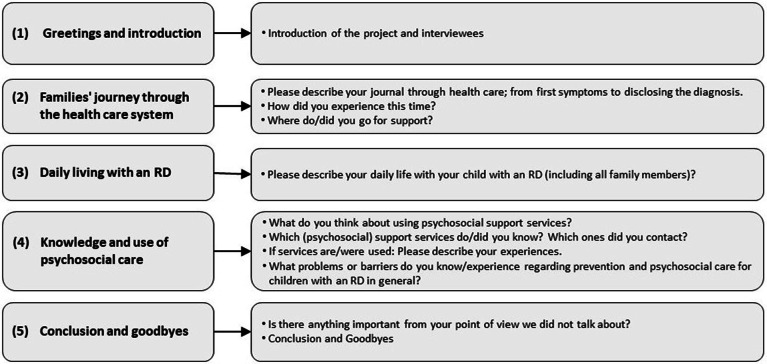
Interview guideline for parents (questions for children were adapted to more accessible language)

This guideline follows a priori-defined structure but also allows additional questions and emerging issues in the context of RD. The guide included [1] an introduction of the project and interviewees, then focused on [2] the families’ journey through the health care system after receiving a diagnosis, [3] daily living with an RD, and [4] knowledge and use of psychosocial care, patient organizations, and support groups (Fig. 1). We translated the questions into a more accessible language for the interviews with children; we addressed the same topics as in the interviews with parents.

Due to its open design, the qualitative approach enables greater insight into the personal view of the respondents [35, 36]. Qualitative interviews capture unexpected information that can be registered on different levels of meaning [37]. This way, the respondents’ impressions and perspectives on the topic can be reproduced.

In an upstream survey, we interviewed professionals working with children and adolescents with RD to gain a comprehensive understanding of all stakeholders involved in caring for pediatric patients with RD and their families [38]. In this study, we report the results of the interviews with affected children and parents of children with RD. The ethical reviews board of the Hamburg Medical Association approved the study before the study started (PV5749). This study presents the methods according to the consolidated criteria for reporting qualitative research (COREQ) checklist.

### Participants and sampling

We aimed to recruit n = 30 children, adolescents, and young adults (8 to 21 years) with a diagnosis of an RD and n = 40 parents of children with an RD (0–17 years) via the cooperating centers [34]. The sampling aimed to follow a stratified recruiting strategy regarding the children’s age (n = 10 children aged 8 to 12 years, n = adolescents aged 13–17 years and n = 10 young adults aged 18 to 21 years, n = 10 parents of children aged 0–4 years, n = 10 parents of children aged 5–7 years, n = 10 parents of children aged 8–12 years and n = 10 parents of children aged 13–17 years).

Parents who agreed to participate in the main study were informed using written and oral information in the participating centers. The parents’ written declarations of consent to participate in the partial study were forwarded to the University Medical Center Hamburg-Eppendorf (UKE). An appointment for the interview was arranged by telephone.

Inclusion criteria for participation in the telephone interview were: [1] that the parents have a child aged 0–17 years with a diagnosis of an RD and [2] have sufficient knowledge of German to participate in a telephone survey. Families with acute psychiatric or somatic treatment needs were excluded from the study.

Children were included in the partial study if [1] they were between 8 and 21 years old, [2] had an RD, and [3] had neither linguistic nor mental impairments. We failed to recruit sufficient pediatric patients via the cooperation centers with these inclusion and exclusion criteria. We broadened our recruiting strategy because of the diagnosis in early childhood or associated comorbidities such as mental or cognitive impairments. We used both the CARE-FAM-NET project and an online call. The written declarations of consent were sent to the UKE for children recruited through the CARE-FAM-NET project and their parents. The online call was published on the website of the Alliance for Chronic RD e.V (Achse e.V., www.achse-online.de) and the online presence of the CARE-FAM-NET project (www.carefamnet.org). For the online presence, the children registered directly via e-mail; after that, a declaration of consent, including an age-appropriate information letter and a pre-paid envelope, was sent by post. When children were younger than 17 years, a written declaration of consent was obtained from their parents.

### Data collection

We conducted the qualitative interview between June 2019 to February 2021. The subject area is narrowed down using guideline-supported telephone interviews, where the most important contents are considered. In addition, with larger numbers of interviews, the statements can be compared [37]. At the beginning of the telephone interview, the respondents were informed about the content of the study. In addition, the subject of the interview was introduced, and information was provided about the aspects of data protection law. Then the respondents were asked to introduce themselves and report their family situation briefly. Questions about the disease and the previous path through the health care system followed the free explanations. The interviewees were then asked about psychosocial care offers and desirable and conceivable support offers. Actual and potential obstacles and barriers were also discussed. Parents were also asked to describe their expectations for the CARE-FAM-NET study. In addition, due to the pandemic, the children were asked about their daily life regarding SARS-Cov-2.

### Data analysis

The interviews were digitally recorded and transcribed using F4 (www.audiotranskription.de). The interviewees were pseudonymized during the transcription by replacing their names with code numbers or letters. Other names mentioned in the conversation were also replaced by letters. Dots symbolized short pauses in brackets, with longer pauses in seconds. The particular emphasis on individual words was recorded using capital letters. The statements have been given time stamps.

**Fig. 2:**
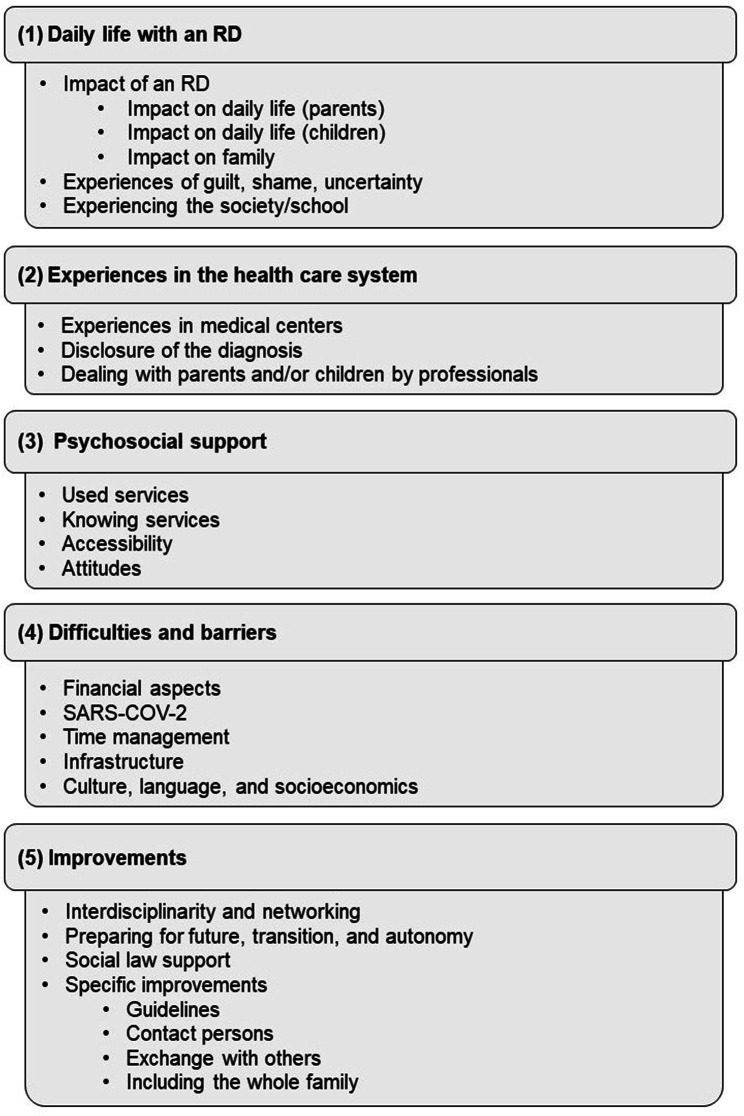
Final coding system

After the transcription, we used the method of focused interview analysis [35, 36] to analyze the qualitative data. Focused interview analysis makes it possible to focus specifically on certain topics or issues and gain detailed insights into the interviewees’ views, experiences, and perspectives. It aims to gather and understand specific information on a particular topic. The researcher focuses on pre-determined questions and searches for specific information in the interview material [35, 36]. We used the software MAXQDA (Version2020) to support the analysis process, which allows for structuring and systematizing large amounts of text [35, 36]. A coding guide was developed, including deductive and inductive categories. Coding rules defined all categories and were illustrated by a representative anchor example [39]. Initially, we used the interview guideline to provide a preliminary framework of deductive (basic coding). Significant sections of the interview text were assigned to the respective categories. The coding guide was inductively supplemented and optimized with additional categories and sub-categories (detailed coding) in the analysis. The team discussed these adjustments until the coding guide was applied to the parents’ and children’s interviews (Fig. 2). In order to minimize subjective effects in the coding, 20% of the interviews were coded by an independent rater (“second-rater”) using the guidelines. We defined an intercoder agreement of at least 70% as an acceptance criterion.

All interviews were conducted in German, transcribed in German, and analyzed in German. For this publication, native-speaking colleagues translated the quotes, which are intended to illustrate the identified codes.

## Results

### Sample description

We recruited a total of 80 parents, including a signed consent form. Four of them could not be reached via telephone to make an appointment. Due to the defined inclusion and exclusion criteria, two interviews with parents were excluded from the analyses because their children were older than 17 (Table 1). So the final sample size of parents consists of n = 74 for further analysis. Most (47%) of the parental interviews were conducted in the age group 0–4 years (n = 35). In the 13-17-year-olds, with n = 7 parental interviews, we did not achieve the target sample size of n = 10. Of the targeted n = 30 interviews with children, a total of n = 15 were conducted (Table 1).

**Table 1:** Final sample size of interviews with children diagnosed with an RD and parents of children diagnosed with an RD

Age groups	0–4 yrs	5–7 yrs	8–12 yrs	13–17 yrs	18–21 yrs	total
parents	35	16	16	7	(2)	**74** (+ 2)
children	-	-	6	5	4	**15**

Of the 74 interviewed parents, 63 were mothers, and 11 were fathers. The parents’ age varied from 23 to 58 years (M = 37.5). On average, the parents had more than one child (1.85 children per participating parent). The range varied from 1 to 5 children. One mother had two children with an RD. Most (81%) parents surveyed were married or lived with a partner. For almost 49% of the participating families, the diagnosis took longer than one year (M = 3.3). Of the 15 interviewed children, 9 participants were male, and six were female. The age of the children surveyed ranged from 8 to 21 years (M = 13.6) (Table 2).

**Table 2:** Sample characteristics

sample characteristics			parents				children		
			n		**%**		n		%
female			63		85.1		6		40
male			11		14.9		9		60
age (in years)			37.5		-		13.6		-
co-habitant parent			60		81		-		-
single parent			14		19		-		-
number of children			1.85		-		-		-
time of initial diagnosis (age of the child)	pregnancy		8		10.8		-		-
	< 1 year		30		40.5		7		46.7
	≥ 1 year		36		48.7		8		533
duration of the interview (in minutes)			19.95				20.6		-

The children’s diagnoses showed a broad range. The major part of the diagnosis was located in the sections of Congenital malformations, deformations, and chromosomal abnormalities (Q00-Q99), Malignant neoplasms (C00-C97), and Metabolic diseases (E70-E90) according to the International Statistical Classification of Diseases and Related Health Problems 10th Revision (ICD-10) [40]. Further diagnoses were located in the sections Neoplasms of uncertain or unknown behavior (D37-D48), Other diseases of blood and blood-forming organs (D70-77), Disorders of other endocrine glands (E30-E35), Episodic and paroxysmal disorders (G40-G47), Haemorrhagic and hematological disorders of fetus and newborn (P50-P61), Diseases of Liver (K70-K77), Extrapyramidal and movement disorders (G20-G26), and Disorders of psychological development (F80-89).

The average length of the interviews was around 20 min. The longest interview had 48 min; the shortest duration was 7 min.

### Intercoder agreement

The second rater coded 20% (n = 18) of randomly selected interviews using the coding guidelines created by the first coder. Based on the number of interviews conducted, 15 parent interviews and three child interviews from all age groups were selected. We ensured that the interviews included parents from different centers when selecting the interviews. We checked the codings regarding the interrater agreement using MAXQDA, and the first check showed an agreement of 77%. We discussed the coding to specify the coding guideline and precise definitions. After that consensus discussion, we coded the selected interviews again and achieved an agreement of 87%.

### Category system

Five main themes emerged from the qualitative analysis of the interviews with pediatric patients and parents describing [1] daily life with an RD, [2] the experiences with the health care system, [3] used psychosocial support, [4] difficulties and barriers, and [5] improvements for patient-oriented support.

### Daily life with an RD

Patients and parents reported intensive effects of the RD on their daily life. Parents had to restructure their daily life. Often the daily life of the family needs to be organized in detail. At the same time, parents and patients reported on necessary precautions and their integration into daily life routines.

*“Monitors initially monitored my son, so when I went out with him, I always had to take everything with me. That means in detail: monitor, resuscitator, oxygen bottle, suction device. It was just a bit of a hassle.“(Mother of a 7-year-old child with a metabolic disease, 38 years old)*.

Children and parents were concerned with the physical effects and medical routines. For example, they reported disadvantages in sports or planning regular medication intake.

*“That is part of my day. […] I’ve been taking pills since I was three years old. […] you don’t even need to think about it anymore, as I said. Yes, that’s a ritual every day.“ (Child with pituitary insufficiency, 15 years, female)*.

However, the parents reported that the children would accept themselves and cope well with their illness. Also, young children (8–13 years) described acceptance of their disease.

*“My son knows that he is sick, but he doesn’t see it as a problem. Well, not at all. “(Mother of an 8-year-old child with a metabolic disease, 35 years old)*.

*“I don’t even know what it is like without the disease” (child with a blood disease, 14 years old, female)*.

The adolescents and young adults (13–21 years) reported experiences of massive limitations due to the RD. They explained that they needed support for many everyday things, leading to extreme dependence on others (especially their family). They discussed a lack of independence and personal responsibility in many matters. For example, the lack of mobility is a common problem in their eyes, as they often do not have or are not allowed to get a driver’s license due to the disease.

*“I especially notice it with the tremor, mostly in everyday life in many situations, because it is now very pronounced in me. Um, that I really need help with many things, […]. So many things no longer work independently. “(Young adult with a neurological disease, 19 years old, male)*.

*[…] You just notice what others at the same age have already achieved, are doing, going to work, going out a lot, going out with friends, traveling and going on vacations, all sorts of things and what you can’t do yourself. It’s difficult, so it’s challenging to deal with it, you get used to it, but it annoys and sometimes bothers.“ (Young adults with narcolepsy, 21 years old, female)*.

Some of the children surveyed stated that they would often be absent from school or work for health reasons or attend medical appointments. In some cases, the RD is so engaging that the children cannot continue participating in everyday school life or complete an apprenticeship.

*“So it didn’t work anymore, so I had to break off my training.“ (Young adults with narcolepsy, 21 years old, female)*.

The compatibility of work and everyday life was reported to bean important aspect for the respondents. Caring for a sick child is very time-consuming, so this would become a new full-time job, and they could no longer pursue any or only reduced gainful employment. Due to the reduced working time/employer status and missing vocational training, the RD results in financial problems or financial dependence from the perspective of young adults.

*“You feel like a 21-year-old is just […] so unimportant, I would say. […] I get pocket money from my mother, at 21, at almost 22 years of age […] And that’s a bit, mhh, yes, I mean, I can’t help it that I’m sick. ”(Young adults with narcolepsy, 21 years old, female)*.

Another impact that most interviewees discussed was the effect of the RD on family life. From the interviewees’ perspective, the RD would frequently be at the center of family life and influence the partnership and dynamics within the family. It has often been described that all family members have to consider the child with the RD. The interviewed parents mentioned ignoring and suppressing their needs and desires, harming family life and exceptionally healthy siblings.

*“I know that my siblings also had to adjust a bit because I got sick all the time. And that they now had to be a little more careful. “(Child with a blood disorder, 14 years* old, female).

The majority of interviewees reported psychological effects due to the diagnosis of the RD. They often felt left alone and powerless in their situation. Many interviewed parents stated they experienced fears and excessive demands at the initial diagnosis. In addition, they felt powerless and desperate. One mother reported that the stress was so great that she even had suicidal thoughts.

*“Well, you get this diagnosis and then …, look how you get along with it.“ (Mother of a 5-year-old child with epilepsy, 40 years old)*.

Besides this, the children mentioned they would feel uncomfortable in social contexts because of their disease. They also reported negative feelings from traumatic situations, such as separation from their parents during hospital stays.

*“I notice that when I see an ambulance with a siren […]. A series of pictures in my head, like when I was three years old, in the hospital, and so on. You just imagine that again. That’s really awesome. ”(Child with pituitary insufficiency, 15 years old, female)*.

Many of the interviewees reported isolation and withdrawal from social life. Due to the RD and its associated efforts, the interviewed parents and children lack resources for social contacts. Often they would be asked about the disease by others, making them difficult to explain. Additionally, the parents experienced being stared at because of the children’s disease. Some of the children reported experiences of mobbing and social exclusion, especially in school.

Next to these negative experiences, patients and parents reported that over time and with the support of others - often including patient-organizations - they have come to terms with the RD. They have developed strategies to cope with the challenges and stay positive. Stronger family cohesion, a focus on what is important in life, and increased competence in medical care support this important coping process.

### Experiences with the healthcare system

The interviewees reported long diagnostic processes and hospital stays. They often stated the need for various clinic appointments with physicians, examinations, and tests until they received the confirmed diagnosis. Hospital stays were often associated with difficulties. Parents reported about clinics not being adequately prepared to care for the children with an RD. The parents experienced the clinics often as haphazard. In some cases, there was no proper accommodation for the accompanying parent. The parents were often separated from their children, which was traumatic.

*“So […] I was not allowed to have my child with me at night, I had to leave the clinic at night, it was only allowed to sit on my child’s bed during the day. […] And I didn’t have the opportunity to be with my child, which would have been extremely important for me and my child. “(Mother of a 4-year-old child with heart disease, 39 years old)*.

Interviewees reported ignorant and overwhelmed professionals. In particular, the announcement of the initial diagnosis was described as very stressful. Parents experienced some professionals as unempathetic. From their perspective, these professionals did not take the time to talk or only provided insufficient information about the RD. The interviewees stated that they did not feel taken seriously by some professionals. Parents reported feeling uninformed about the diagnosis and missed psychosocial support, especially after the initial diagnosis.

*“You can’t just send a woman with a small child home [after the diagnosis has been communicated] and say: have a nice weekend. That doesn’t work.“ (Mother of a 1-year-*old child with a genetic defect, 41 years old).

Some parents experienced the psychosocial support from clinical psychologists as inadequate and untrained for these special situations. Parents reported that psychosocial support ended by leaving the hospital.

*“So […] I think the [psychologist] was a bit overwhelmed with what actually happens in such an intensive care unit.“ (Mother of a 1-year-old daughter with a heart defect, 35 years old)*.

Besides these negative experiences, half of the parents reported positive contact with specialists. The parents appreciated professionals showing efforts, availability, and open and honest communication. In addition, some of the interviewed children stated that their doctors, in particular, would serve as their first contact persons and that they could turn to them in confidence if they had any problems. Children, adolescents, and young adults reported that they trust the physicians, experience medical procedures such as blood sampling as patient orientation and get quick responses if needed via e-mail. Because the children visit the doctor more often for medical examinations as healthy children and their RD often makes them special to the physician, there is an excellent and intensive physician-patient relationship from the children’s point of view.

*“So, we always have special appointments, like 10 minutes before opening, for example, if you get a vaccination or what ever. It’s really great. I feel in great hands at the pediatrician and trust him.”* (adolescent, 13 years old).

Nevertheless, most parents reported looking for information and assistance concerning the RD alone. Some of these parents mentioned that they had become experts and would now try to help other parents and families through their acquired expertise.

### Used psychosocial support

Most respondents said they had not been informed (especially by the specialists) about psychosocial care offers or did not know whom to turn to. Some respondents said they would have liked to get help if they had been informed about offers.

Many respondents also stated they felt they had mainly been informed and enlightened by the doctors about the RD, especially regarding medical care. The main focus was contact with doctors during further therapy, but hardly at the time, the confirmed diagnosis was disclosed.

Some parents stated they had no or difficult access to psychosocial care offers. It was repeatedly stated that a need for care on the part of the parents had been addressed. Nevertheless, the supervising specialist staff made no suitable care offer available. Often this was associated with the ignorance of specialists.

They reported an inhibition threshold based on the fear of stigmatization if they would use psychotherapeutic offers.

*“Uh, of course, there is always a slight inhibition threshold, because now you are not called uh, because you are also a little afraid that you might also be labeled there.“ (Father of a 1-year-old child with a metabolic disease, 36 years)*.

A few respondents who had already used psychosocial offers expressed a consistently positive attitude. They would find the supply valuable and helpful. There were also parents for whom it was important that their children would receive psychosocial support in certain phases of life (for example, dealing with the diagnosis).

Parents reported searching for information on the RD and psychosocial support using online-search. They primarily used information and exchange with others via patient-organizations. However, family and friends were also essential in dealing with the disease and its daily challenges. The experiences regarding social pediatric centers (SPZ) vary a lot. While some parents experienced these centers’ support as very helpful, others reported not receiving psychosocial support. Some interviewees described positive experiences with psychologists but also a lack of knowledge of the specific challenges due to the RD.

Parents accepted best and used more often support services that include psychological support, medical support, social law advice, and additional offers for, e.g., siblings that were coordinated by one institution and which work together interdisciplinary with clinics and hospitals.

Almost all families used alternative support. This support included school assistance, a teacher for children with special needs, or social workers. Parents reported getting support in administrative tasks such as forms and applications from these professionals, which they greatly appreciated.

### Difficulties and barriers to using psychosocial support

More than half of the interviewees mentioned barriers to using psychosocial support caused by difficulties in time management. The parents reported multiple time clashes due to combining therapy appointments, household management, family obligations, and caring for other (healthy) siblings. As a result of these time-intensive requirements, parents and children reported a lack of time and capacity to take advantage of psychosocial offers for any support.

*“Um, with us, I think the biggest problem is time and everyday life. We have so many therapeutic appointments for our son and visits to the doctor, etc. It is difficult to include something like that [psychosocial care offers] or take care of them. “(Father of a 1-year-old child with a neurodegenerative disease, 36 years)*.

Many respondents had difficulties using certain therapies, as offers are often unavailable in the immediate vicinity. In particular, the long journeys and the associated effort constitute a significant obstacle. The respondents were often confronted with long waiting times and lists. As a result, certain offers could not have been taken, although they were needed.

A few parents also named linguistic and cultural difficulties as possible and conceivable obstacles. The parents mainly saw difficulties with incorrect or insufficient information, which comes with a language barrier. Parents mentioned that a higher educational background might be beneficial to understand the professionals’ technical terms and get access to psychosocial support. These parents would ask more often and also demand support if needed.

*“Oh, sometimes, yes, I sometimes don’t understand the technical words or what they explain to me.“ (Mother of an 8-year-old child with a genetic defect, 43 years old)*.

In addition, the corona pandemic exacerbated the interviewed parents’ organizational and scheduling problems. On the other hand, the interviewed children found wearing mouth-nose protection and decorum rules a nuisance. However, most reported no worries about their underlying illness in connection with SARS-Cov-2.

### Improvements in patient-oriented support

Most parents and children interviewed would have liked more access to and information about psychosocial offers. Interviewees often mentioned that they would have generally hoped for a reference from the physicians to existing offers.

It was essential to the respondents that a contact person was available to whom one could turn in the event of problems and who could serve as a coordinator and, if necessary, refer them to others.

Above all, support from the time the diagnosis is made is essential to the respondents. For many parents, disclosing the diagnosis was a major turning point in their previous lives. Insecurities and excessive demands characterized the first time, so many respondents would have preferred direct psychological contact and continuous support to prevent psychological stress and helplessness.

*“So everyone, every family who receives such a diagnosis needs first help, care, before the uh, before all this medical stuff even goes on, someone has to be there to say: Hey, I’ll listen to the worries, I listen to the fears, I take my time and I uh can help too. “(Mother of a 1-year-old child with a metabolic disease, 30 years old)*.

Many respondents wished for support and care for other family members. The parents mainly hoped for offers where the entire family would be involved, and family cohesion would be strengthened. For some parents, family-oriented offers were very important. Taking care of the siblings was one of the main topics, and the parents appreciated offers focusing on the sibling and their developmental skills. In the case of the children surveyed, the older children wished for support for their relatives. They often hoped that their parents would be relieved and that they would learn to deal better with their illness.

Above all, however, there should be guidelines for doctors and their caring staff. The treating specialists were often at a loss and overwhelmed with the RD (see health system). A guide with additional addresses of experts, information on the information needs of those affected, and possible therapy offers are essential from the respondents’ point of view.

A few interviewed parents and children wanted a more intensive exchange with other affected persons. They hoped to understand their situation better and get help from those affected who could empathize with their situation through communication with others.

Many interviewees, especially the parents, would like interdisciplinary work with the caring staff. Access to other specialist departments and other offers and therapies should be simplified. They addressed the wish that medical offers and psychological-therapeutic offers should be networked. They hoped that more far-sighted and more comprehensive care would be available. In addition, they promise themselves more time and resources through better cooperation between the specialist departments.

*“But then it would be nice if the doctor could sit down with the psychologist, or that they all form a unit. So directly on-site at the university clinic, that would be great. “(Mother of an 8-year-old child with a metabolic disease, 42 years old)*.

Almost half of the participants expressed difficulties concerning social law aspects. In the beginning, many parents would not have known what aids they would be entitled to or how they could use these aids.

The interviewed parents and children described that many uncertainties shaped their future. Above all, the children wanted support with the transition from school to training or studies and support when entering the world of work. In particular, independence and independence are crucial to some children surveyed.

## Discussion

Rare diseases pose significant challenges to affected individuals and their families, often leading to a wide range of daily life changes. These necessary changes can profoundly affect various aspects of family dynamics, including emotional well-being, financial stability, social interactions, and overall HRQOL.

The interviewed parents and children stated that the RD strongly affects their everyday life, including various limitations. RD and its consequences lead to reorganization, changes in family functioning, and reduced spontaneity [41–43].

Caring for a child with an RD can impose a substantial emotional burden on parents and other family members, e.g., healthy siblings. Parents often experience high levels of stress, anxiety, and depression as they navigate through the complexities of managing their child’s condition [44]. Parents of children with RD reported higher levels of psychological distress than those of typically developing children [16]. This emotional burden can affect marital relationships, parent-child interactions, and overall family cohesion.

The families often face social isolation due to the unique demands and challenges they encounter. The complex medical needs of their child, combined with limited knowledge and awareness of the rare condition within the community, can result in a lack of understanding and support from friends, extended family, and even healthcare professionals. This isolation can lead to feelings of loneliness, exclusion, and a reduced sense of belonging.

In the interviews, the respondents reported that they isolated themselves due to the RD and barely participated in social life. The disease is easier to control at home, and one escapes social prejudices. Parents mentioned that they were “looked at differently” in public or asked about their child’s illness, making them uncomfortable. The interviewed children with RD also described that they had experienced increased exclusion because of their RD. Especially children with visible manifestations of the disease reported that they would withdraw from public life because of negative comments. Also, previous studies found that those affected by RD isolate themselves from the social environment [27, 43, 45]. Therefore, it is essential to support families to adapt and learn appropriate coping strategies to deal with societal prejudices [45]. Additionally, it is helpful to enlighten society regarding RD to reduce prejudice and enable inclusion [46].

The daily life impairments faced by families with children having RD can have a cumulative effect on their overall HrQoL. Constantly juggling medical appointments, caregiving responsibilities, and managing emotional and financial burdens can lead to decreased well-being and diminished life satisfaction. Parents of children affected by RD reported lower HrQoL than the general population [47]. While physical HrQol was mainly reported to be comparable with parents of healthy children or reference values, mental HrQoL was reported to be significantly reduced [48]. Interventions aimed at improving the HrQoL of these families should focus on comprehensive support systems that address the multifaceted challenges they face.

A significant challenge is the fragmentation of care. RD often require multidisciplinary management involving multiple healthcare providers and specialists. Parents (and patients) may find it difficult to coordinate appointments, manage different treatment plans, and communicate effectively with different healthcare providers [49]. This fragmentation can lead to a lack of continuity of care, increasing the burden on parents and potentially compromising the overall quality of care for the child.

Pediatric patients may experience physical discomfort, pain, and frequent hospitalizations, disrupting their normal routines and activities [50]. This can result in feelings of isolation, reduced social interactions, and challenges in educational settings. The interviewed children reported that their school day was burdened by absences due to illness or needing medical appointments. The children are often absent from school, making it difficult to follow the lessons. Therefore, teaching should be individually adapted to children with RD and designed flexibly (for example, online learning opportunities and tutoring services) to give children with RD equal educational opportunities [51].

The interviewees often mentioned that the focus of family life was predominantly on the child’s RD. All family members had to consider the special needs of the child with the rare chronic health condition, which meant that siblings might be neglected or the partnership of the parents suffered. The influence of an RD on the development of healthy siblings is described in many studies [43, 52–55]. Therefore, any support should also address the siblings’ needs. The families’ handling of the RD in daily life depends on communication and coping strategies within the family [22]. Accordingly, good family cohesion, good and regular communication between family members, and a positive attitude can help cope with the RD [56].

The financial impact of RD can be overwhelming for families. Families with children having RD frequently face higher medical expenses, including specialized treatments, medications, and assistive devices. Moreover, the need for frequent doctor visits, hospitalizations, and therapy sessions can lead to additional costs such as transportation and accommodation. Families with children with RD faced this substantial economic burden, often resulting in financial instability [27, 42]. The interviewed parents often reported restructuring their careers or partially giving up their jobs because of the new caregiving task. Especially mothers would give up their jobs to care for the sick child [42, 43, 57].

Disclosing a diagnosis of an RD is a delicate and complex task, as it has profound implications for both the child and the parents. The interviewed parents and children reported a long and complex diagnostic process, including numerous physician visits, examinations, and extended hospital stays. Our sample’s average time to the initial diagnosis was around 3.3 years. This long period from the first symptoms to the confirmed diagnosis dramatically burdens patients and their families [6, 58–60]. This period is characterized by multiple medical consultations, tests, and procedures, which can be distressing for the child and their family [61]. The prolonged uncertainty and invasive investigations may contribute to anxiety, fear, and emotional distress in pediatric patients. Parents of children with RD often describe a delayed diagnosis, resulting from a lack of awareness and knowledge about RD among healthcare professionals [62]. This delay can lead to frustration, anxiety, and a prolonged period of uncertainty for parents, as they seek answers and appropriate care for their child.

The experience of receiving a diagnosis of an RD for their child can be overwhelming for parents. Initially, parents may experience a range of emotions, including shock, disbelief, anger, guilt, sadness, and anxiety [63]. They may struggle to comprehend the disease’s rarity and potential severity, leading to adjustment and emotional turmoil. This reaction is similar to that observed in parents of children with more common chronic illnesses [64].

Parents often face challenges in understanding the disease, finding appropriate medical care, and accessing necessary resources. The lack of available information and limited expertise in the medical community regarding rare diseases further compounds these challenges. Parents may also feel isolated, as the rarity of the condition can result in limited support networks and difficulty connecting with other families facing similar circumstances [65]. Parents reported being left alone after the disclosure and missing a contact person for any further questions. Many parents experienced the professionals as unempathetic and distant [43, 66–68]. Patients and their parents could deal better with limitations and restrictions in daily life due to a health condition if professionals confirm the diagnosis and, at the same time, offer a disease-specific treatment concept [69].

Healthcare professionals are critical in supporting parents and patients after disclosing an RD diagnosis. Providing accurate and comprehensive information about the disease, its prognosis, available treatments, and ongoing research can help parents and patients better understand and cope with the situation [70]. Clear communication is vital, and healthcare providers should use non-technical language, be empathetic, and allow parents to ask questions and express their concerns [63]. In addition to information, parents and patients require emotional support. Establishing a trusting and supportive relationship between healthcare providers and the family is essential. Encouraging them to express their emotions and concerns without judgment can help alleviate some of the emotional distress associated with the diagnosis. Referrals to mental health professionals or support groups specialized in RD can provide additional avenues for emotional support [63, 71]. In the interviews, parents described that professionals did not take the time to disclose the initial diagnosis, and the parents did not remember detailed information about the disease, treatment options, or psychosocial support offers. Parents experienced professionals as uninformed, resulting in delayed diagnoses [68].

Physicians did not know about the specific health condition from the parents’ perspectives. They referred the families to specialists too late [72]. More information about specialized centers and better networking are necessary in order to be able to diagnose an RD as early as possible. The National Action Alliance for People with RD (NAMSE) was founded nationally in Germany. The European Reference Network (ERN) at the European level also contributed to improved care [73, 74]. These projects must be expanded and promoted to gain knowledge to support children with RD and their families sufficiently.

Approximately 25% of physicians reported being overwhelmed in interaction with parents [67], highlighting the need for specialized professional training. Distant conversations without sufficient information lead to a psychological burden for the parents [41]. Parents wish to be integrated into the treatment decisions based on understandable information, resulting in better health status of the affected children [75, 76]. Parents of children with chronic illnesses are often not informed sufficiently or much too late about psychosocial services [27, 66]. This aggravates the psychological symptoms. Most interviewed parents mentioned feeling an intense mental burden due to the RD. Feelings of powerlessness, loneliness, despair, and being overwhelmed were frequently described. These findings are consistent with studies investigating the mental impact on parents of chronically ill children [5, 27, 43]. One reason for the late provision of information on psychosocial services may be that physicians sometimes recommend psychotherapeutic services too late or not at all, as this appears to be encroaching and impolite [77]. Often this aspect is connected with the social stigmatization of psychotherapy. This social stigmatization can lead to bias/shame among those affected, which hinders them from seeking psychological support [78]. Therefore, extensive education about psychotherapeutic/psychosocial offers is essential to minimizing these inhibitions. In addition, social awareness should be strengthened to reduce social prejudices and stigmatization [79].

The interviewees wished for psychosocial support, especially in the context of disclosing the initial diagnosis, to prevent psychological stress. The period between the first signs of mental stress and the appropriate therapy is often too long [80]. This period would need to be shortened to reduce an increase in mental health symptoms [80]. In addition, it would be desirable if psychosocial care at diagnosis is part of routine care and regularly offered [68, 81].

The interviewees expressed the need to take the initiative to find support offers and gather the most critical information on RD on their one [30, 43, 66]. Furthermore, many interviewees, especially the parents, reported on the advantages of psychosocial services due to a lack of time [17, 18]. Parents’ primary goal is to ensure the care of their children. They often cannot integrate personal psychosocial support into daily life due to limited time capacities or estimate the child’s medical care as more important than parental psychosocial support.

Families often cannot utilize support offers due to infrastructural barriers, such as long distances. Interviewees from more rural areas complained that long distances were a severe obstacle. The care landscape provides too few therapists, whose use is usually accompanied by a long waiting time and precludes spontaneous counseling [82, 83]. As a result, families sometimes have to wait months for psychological therapies, even though there is an acute need for professional support.

Parents and patients may benefit from assistance navigating the healthcare system and accessing appropriate resources. Healthcare providers can play a crucial role by connecting families to relevant support organizations, educational materials, financial assistance programs, and community services. Facilitating communication between families with children affected by the same rare disease can provide a sense of community and mutual support.

### Implications for practice and research

Psychosocial support is crucial in addressing the unique challenges faced by families dealing with RD. Providing comprehensive psychosocial support to parents, pediatric patients, and siblings can improve emotional well-being, enhance coping mechanisms, and promote overall family resilience.

Parents of children with RD often experience high levels of stress, emotional distress, and social isolation. Psychosocial support can help address these challenges and improve parental well-being. Support groups and counseling services allow parents to connect with others facing similar circumstances, share experiences, and gain emotional support [84]. These interventions foster a sense of community and reduce feelings of isolation. Additionally, providing accurate and accessible information about the rare disease, its management, available resources, and coping strategies is vital [85]. Parental education programs and workshops can empower parents to navigate the healthcare system, advocate for their child, and make informed decisions regarding treatment options [86]. By equipping parents with knowledge and skills, psychosocial support enhances their ability to cope effectively with the demands of caring for a child with an RD.

Psychosocial support is equally important for pediatric patients with RD. These children often face unique challenges related to their physical health, emotional well-being, and social interactions. Supportive interventions should provide age-appropriate outlets for expression, reduce anxiety, and promote psychological resilience [87]. Involving pediatric patients in support groups or peer mentoring programs allows them to interact with peers who share similar experiences. These interactions can enhance their social skills, self-esteem, and overall well-being [5]. Creating safe spaces for open communication and emotional expression helps pediatric patients navigate the emotional impact of their condition and fosters a sense of belonging.

Siblings of pediatric patients with RD are often neglected in research and clinical practice. They also experience challenges and emotional strain. They may feel neglected, resentful, or overwhelmed due to the family’s focus on the affected child [88]. Providing psychosocial support tailored to the needs of siblings can mitigate these challenges and foster healthy sibling relationships [89]. The psychosocial needs of siblings of children with an RD should also be considered when treating or caring children with an RD. Sibling support programs offer opportunities for siblings to engage with peers who face similar circumstances, share their feelings, and learn effective coping strategies [90]. These interventions promote empathy, understanding, and support among siblings, reducing feelings of isolation and improving their overall well-being.

Healthcare professionals can enhance emotional well-being, improve coping mechanisms, and foster overall family resilience by providing comprehensive support to parents, pediatric patients, and siblings. Support groups, counseling services, educational programs, and sibling support interventions are essential components of psychosocial support programs for families dealing with rare diseases.

### Strengths and limitations

The distribution of the age groups varied considerably. In some age groups, the target sample could not be reached. This may be related to the fact that most RD are congenital, and the diagnosis is often made in childhood. Parents’ psychological stress is most incredible immediately after the initial diagnosis. In contrast, parents of older children or adolescents with RD, have already developed strategies for dealing with RD over the years. As a result, the affected person may feel less or hardly any psychological stress [12, 13, 22]. In the interviews with children, adolescents, and young adults with RD, we did not reach the planned case number of n = 30. The final analyses of these qualitative data showed a saturation in that no new themes [91] and hardly any new codes [92] were identified and added. Nevertheless, we must assume - especially against the background of the heterogeneity of RD - that further themes among children, adolescents, and young adults remained undetected. Therefore, the results for children, adolescents, and young adults should be interpreted cautiously.

Furthermore, it became apparent that mothers predominantly attended the interviews (n = 63, 85%). These are often the family caregivers and feel more burdened by the RD of the child than the fathers [17, 18, 43]. It is also mainly the mothers who cope with the disease within the family. At the same time, fathers are usually not involved like mothers in family management [42].

## Conclusions

RD represent a major challenge for all family members involved – patients, parents, and siblings. The experiences of parents and affected children in daily life, medical and psychosocial care show a need for target-group specific support, including training of health care professionals, low-threshold access care services, and practical help for all family members.

## Data Availability

The datasets generated and/or analyzed during the study are publicly unavailable. This was done to keep the interview transcripts confidential.
